# *Ozoroa insignis reticulata* (Baker f.) R. Fern. & A. Fern. Root Extract Inhibits the Production of Extracellular Proteases by *Staphylococcus aureus*

**DOI:** 10.1155/2021/5599129

**Published:** 2021-10-29

**Authors:** Jonathan Katsukunya, Rumbidzai Makurira, Stanley Mukanganyama

**Affiliations:** Department of Biotechnology and Biochemistry, University of Zimbabwe, P.O. Box MP 167, Mt. Pleasant, Harare, Zimbabwe

## Abstract

Treatment of infections caused by *S. aureus* has become a challenge due to the emergency of resistant strains. *Ozoroa reticulata* root extracts have been used in traditional medicine to treat throat and chest pains in Zimbabwe. The objective of the study was to determine the effects of *O. reticulata* root bark extracts on the production of extracellular proteases by *S. aureus*. The root barks were collected, dried, and crushed into powder. To obtain different phytoconstituents, plant extractions were performed. Extractions were carried out using two solvent mixtures: ethanol : water (50 : 50 v/v) and dichloromethane : methanol (50 : 50 v/v). Serial exhaustive extractions were also performed using methanol, ethanol, dichloromethane, acetone, ethyl acetate, hexane, and water. The broth microdilution assays were used to assess the antibacterial effects of the *Ozoroa reticulata* root bark extracts against *S. aureus*. Ciprofloxacin was used as a positive control. Qualitative screening for extracellular protease production by *S. aureus* on BCG-skim milk agar plates using the most potent extract was carried out. The proteolytic zones were measured and expressed as the ratio of the diameter of the colony to the total diameter of the colony plus the zone of hydrolysis (*P*_*z*_ values). The ethyl acetate extract was found to be the most potent inhibitor of the growth of *S. aureus* with 99% inhibition and a minimum inhibitory concentration (MIC) of 100 *µ*g/mL. Inhibition of extracellular protease production was directly proportional to the concentration of the extract. At 100 *µ*g/mL, the ethyl acetate extract had a *P*_*z*_ value of 0.84, indicative of mild proteolytic activity. A *P*_*z*_ value of 0.94 was observed at a concentration of 200 *µ*g/mL and signified weak proteolytic activity. In conclusion, the extract inhibited the production of extracellular proteases in *S. aureus*. Further work on the isolation and purification of bioactive compounds responsible for inhibiting the production of extracellular proteases is of importance in the discovery of agents with antivirulent effects on *S. aureus*.

## 1. Introduction

Bacterial infections have emerged to be among the biggest threats in the 21^st^ century due to the emergence of antibiotic-resistant strains of bacteria. This has led to difficulties in treating common bacterial infections [1]. Among the most serious threats are infections that are caused by drug-resistant *Campylobacter*, *Enterobacteriaceae*, *Enterococcus*, *Pseudomonas aeruginosa*, *Shigella*, *Salmonella typhi*, and *Staphylococcus aureus* [2]. *S. aureus* is a pathogen of medical concern because of its intrinsic virulence [3]. The pathogenesis of *S. aureus* is mediated by several virulence factors. The factors include protein A, *α*-haemolysin, extracellular adherence protein, chemotaxis inhibitory protein, enterotoxins, toxic shock syndrome toxins, and the production of extracellular enzymes [4, 5]. Extracellular enzymes are a different type of enzymes that are used by bacteria for invasion. These include coagulases, hyaluronidases, lipases, staphylokinases, phospholipases, deoxyribonucleases, and proteases [6].

*S. aureus* can cause a variety of severe infections and adjust to diverse ecological settings. In Zimbabwe, *S. aureus* has been associated with a number of outbreaks, including food poisoning [7]. In many developing countries, the occurrence of food-borne diseases is often coupled to resistant bacteria [8]. Methicillin-resistant *Staphylococcus aureus* (MRSA) has shown resistance to drugs commonly used in Zimbabwe [9, 10]. This is mainly pertinent to Critical Care Units (CCUs) and theatres. From patients admitted to CCUs in one of the referral hospitals in Zimbabwe, 94% of the MRSA isolates showed multidrug resistance [11]. Bacteria that show increased resistance against antimicrobial agents and form biofilms are of great medical concern. MRSA strains form more biofilm than Methicillin-susceptible *Staphylococcus aureus* (MSSA) strains [12]. Biofilm-mediated infections produce a poor prognosis for patients. Therefore, there is a need to search for new strategies to prevent and treat biofilm-mediated infections as well as reducing the invasiveness of the bacteria *in vivo*.

Plants possess defense mechanisms against invading pathogens. These include the production of biologically active compounds known as phytochemicals. Phytochemical extracts have been reported to have a biological activity such as anticancer, antimicrobial, antioxidant, antidiarrhoeal, analgesic, and wound healing activities [13]. This makes them attractive as alternative therapeutic options against several disorders [13]. Several plants have been used traditionally to treat several bacterial infections and this knowledge has provided the need for studies into the antimicrobial activities of different plant extracts [14]. One of the plants that are widely used traditionally as a remedy for different ailments and infections is *Ozoroa insignis reticulata* [15]. Most traditional healers in Zimbabwe use the leaf and bark macerate or infusion of this plant to treat diarrhoea, kidney and liver complaints, ulcers, throat infections, chest pains, and schistosomiasis. In some instances, a paste of leaves and bark is usually applied to the skin to treat several skin diseases and infections. Infusions of the root extracts are usually taken orally by women after childbirth to increase lactation [16]. The current study aimed to determine the effects of the root bark extract on the growth and extracellular protease production in *S. aureus*.

## 2. Materials and Methods

### 2.1. Reagents and Materials

All chemical reagents used in this study were purchased from Sigma-Aldrich (Darmstadt, Germany). Hexane, DCM, acetone, ethyl acetate, methanol, and ethanol used for extractions were of reagent grade and were distilled before use. Nutrient agar media (1 g/L meat extract, 2 g/L yeast extract, 5 g/L peptone, 5 g/L sodium chloride, 15 g/L agar, pH 7.4 ± 0.2 at 25°C) and nutrient broth media (15 g/L peptone, 3 g/L yeast extract, 6 g/L sodium chloride, 1 g/L D-glucose, pH 7.5 ± 0.2 at 25°C) were used as growth and enrichment media, respectively. Ciprofloxacin and 3-(4, 5-dimethylthiazol-2-yl)-2, 5-diphenyltetrazolium bromide (MTT) were also purchased from Sigma-Aldrich (Darmstadt, Germany).

### 2.2. Plant Collection and Preparation

The root barks of *Ozoroa reticulata* voucher number BR1 F1 used in this study were obtained from an urban woodland in Richmond, Bulawayo, Zimbabwe (20°5′6″ S; 28°33′7″ E) in November 2018. Ethnobotanical surveys were carried out by Ms. R. Makurira (Department of Biotechnology and Biochemistry, University of Zimbabwe) who interviewed 9 traditional healers in Bulawayo Province (Zimbabwe) leading to the identification *Ozoroa reticulata* (Baker f.) R. Fern. & A. Fern. Taxonomical authentication of the identity of the plant was performed by a botanist at the National Botanical Gardens of Zimbabwe in Harare. The root barks were washed, dried in an oven at 45°C, and ground to a fine powder using a mechanical grinder. For total extraction, the powdered sample was soaked in two mixture solvents: 50 : 50 (v/v) DCM: methanol and 50 : 50 (v/v) ethanol: water. For serial exhaustive extraction, seven solvents with different polarities were used to macerate the powdered sample. The solvents were hexane, dichloromethane, ethyl acetate, acetone, ethanol, methanol, and water. After the maceration period, all mixtures were clarified by filtration, using a Whatman filter paper No. 1 (Sigma-Aldrich, Darmstadt, Germany). The filtrates were allowed to air dry under a fan at room temperature. The dried filtrates were then scraped, weighed, and stored for later use.

### 2.3. Antibacterial Test

The antibacterial activity of the extracts was assessed using the broth microdilution method [17]. The root bark extracts were tested against *Staphylococcus aureus* NCTC 6571 (Culture Collections, Public Health England, Salisbury, UK). Stock solutions were prepared by dissolving the extracts in dimethyl sulfoxide (DMSO). Two‐fold dilutions of the extracts in nutrient broth were dispensed in a 96-well microplate. Overnight liquid cultures of *S. aureus* cells were adjusted to 0.5 McFarland's standard to make a final concentration of 2 × 10^6^ CFU/mL. The inoculum was placed in each well and incubated at 37°C for 24 hours in an incubator (Lab Doctor^TM^, MIDSCI Co., Valley Park, USA). Ciprofloxacin was used as a positive control while a mixture of nutrient broth and DMSO was used as a negative control. The bacterial suspension without extracts was used as the growth control. Preincubation readings were recorded at 590 nm using a Genios Pro microplate reader (Tecan Group Ltd. Zurich, Switzerland). Postincubation readings were recorded after 24 h and the cell viability test was carried out by the MTT assay. The assay involved the addition of the MTT dye to each well, incubation for at least 2 h, and then observation of colour changes.

### 2.4. Determination of Minimum Inhibitory Concentration and Minimum Bactericidal Concentration

To determine the MIC of the most potent extract, a stock solution of 400 *µ*g/mL of the extract was prepared. Two‐fold serial dilutions were done in a 96-well microplate using nutrient broth to obtain the following concentrations: 200, 100, 50, 25, 12.5, 6.25, 3.125, 1.562, and 0.781 *µ*g/mL. Cells exposed to extracts were incubated at 37°C for 24 h and MIC was determined. The minimum inhibitory concentration (MIC) was determined as the lowest concentration of the extract at which no visible growth of *S. aureus* was observed. To determine the MBC of the most potent extract, inoculations from the wells with half MIC, MIC, and twice MIC were inoculated on a nutrient agar plate. The plate was incubated at 37°C and observed after 24 h. The concentration at which no visible growth of *S. aureus* was observed was noted as the MBC.

### 2.5. Screening for the Production of Extracellular Proteases

Screening for the production of extracellular proteases was done by a method for the detection of protease activity on agar plates described by Vijayaraghavan and Vincent [18] with some modifications. Overnight cultures of *S. aureus* cells were standardised to make a final concentration of 1 × 10^6^ CFU/mL. A nutrient agar stock mixture of 200 µg/mL of the most potent extract was prepared along with 1% (w/v) skimmed milk and 0.0015% (w/v) bromocresol green (BCG). A Two‐fold serial dilution of the nutrient agar stock mixture was performed to obtain a 100 *µ*g/mL nutrient agar mixture of the same extract. These were poured onto two separate Petri dishes and allowed to set. The third Petri dish contained the BCG-skim milk agar prepared without the extract. For protease activity screening, 5 *µ*L of the standardised *S. aureus* cells were placed on all the BCG-skim milk agar plates prepared and incubated at 37°C in an incubator. The plates were observed after 24 hours for any zones of proteolysis. The proteolytic zones were measured, recorded, and expressed as *P*_*z*_ values according to the following equation:

(1) $$\begin{array}{c} P_{z}=\frac{\left(\text{colony diameter}\right)}{\left(\text{colony diameter}+\text{ zone of clearance}\right)}. \end{array}$$

### 2.6. Statistical Analysis

Analysis was performed using GraphPad Prism for Windows, version 8.4.3 (GraphPad Software, San Diego, California, USA). Statistical analysis of the results was done using one-way ANOVA. This was followed by Bonferroni's multiple comparison post tests and values of *P* < 0.05 or less were considered significant.

## 3. Results

### 3.1. Plant Extractions

The percentage yields for total and serial exhaustive extractions performed on root barks from *O. reticulata* were determined as presented in Table 1. The percentage yields are expressed as the ratio of the dry weight of plant extract to the weight of the plant material used for the extraction process. The highest percentage yield was obtained from the ethanol: water extract (15%) followed by the methanol extract (10%). The lowest percentage yield was obtained from the water extract (2%). The colour and consistencies of the root bark extracts after drying are shown in Table 1. All root bark extracts had a brown colour except for the hexane extract which was pale yellow. The ethyl acetate and hexane extracts were viscous solids while all the other extracts were solid.

### 3.2. Antibacterial Activity of *O. reticulata* Root Bark Extracts

**Figure 1:**
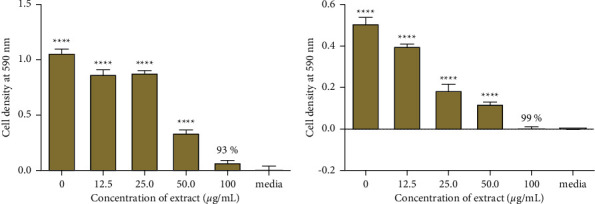
Antibacterial effects of *O. reticulata* root bark extracts against *S. aureus.* The data are obtained by incubating *S. aureus* cells in the presence of (a) DCM extract and (b) ethyl acetate extract. All values are expressed as the mean ± SD of four replicates. Statistical one-way ANOVA and Bonferroni's multiple comparison test (where ^*∗*^ < 0.05, ^*∗∗*^ < 0.01, ^*∗∗∗*^ < 0.001, ns = no significant difference vs. media) show that there is no significant difference between media and cells exposed to 100 *µ*g/mL of ethyl acetate extract. The percentage inhibitions of growth of *S. aureus* by the DCM and ethyl acetate extracts were 93 and 99%, respectively.

The percentage inhibition of growth for the 9 extracts of *O. reticulata* root barks against *S. aureus* (NCTC 6571) is shown in Table 1. Typical results for the determination of the percentage inhibition of growth are shown in Figure 1. A decrease in optical density (OD) at 590 nm was observed as the concentration of the extract increased. The most potent extract was the ethyl acetate extract with a percentage inhibition of 99%. This was followed by the DCM extract with 93%. The least potent extract was the DCM: methanol extract with a percentage inhibition of 29%.

### 3.3. Determination of the Minimum Inhibitory Concentration and Minimum Bactericidal Concentration

**Figure 2:**
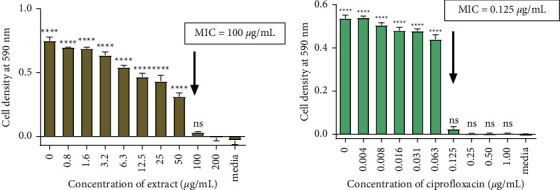
The minimum inhibitory concentration of (a) ethyl acetate extract and (b) ciprofloxacin. All values are expressed as the mean ± SD of four replicates. Statistical one-way ANOVA and Bonferroni's multiple comparison test (where ^*∗*^ < 0.05, ^*∗∗*^ < 0.01, ^*∗∗∗*^ < 0.001, ns = no significant difference vs. media) show that the lowest concentration where there is no significant difference between media and cells is 100 *µ*g/ml. Therefore, the MIC for the extract is 100 *µ*g/mL.

The antibacterial effect of the most potent ethyl acetate extract was investigated at a broader concentration range of 0 to 200 *µ*g/mL. The lowest concentration of the extract that exhibited complete inhibition of growth of *S. aureus* was 100 *µ*g/mL as shown in Figure 2(a). The MIC value of the ethyl acetate extract was greater than that of the standard drug, ciprofloxacin. Ciprofloxacin had an MIC of 0.125 *µ*g/mL as shown in Figure 2(b). The investigation into the bactericidal effects of the ethyl acetate extract against *S. aureus* (NCTC 6571) showed that the extract was bacteriostatic at tested concentrations and, therefore, the MBC was greater than 200 *µ*g/mL.

### 3.4. Screening for the Production of Extracellular Proteases

**Figure 3:**
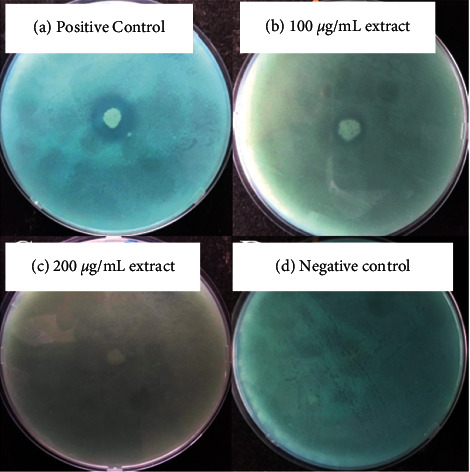
The effects of the *O. reticulata* ethyl acetate extract on the production of extracellular proteases by *S. aureus*. A distinct halo is produced for the positive control (a), less distinct haloes are produced on plates containing 100 *µ*g/mL (b) and 200 *µ*g/mL (c) of the ethyl acetate extract while no growth or haloes are obtained for the negative control (d) after 24 h of incubation.

The production of extracellular proteases by *S. aureus* was detected by the presence of zones of clearance around the colonies on BCG-skim milk agar plates as shown in Figure 3. The sizes of the haloes are proportional to the number of proteases produced. In the presence of the ethyl acetate extract, the sizes of the haloes were smaller as compared to the positive control (no extract). The size of the haloes became less distinct as the concentration of the ethyl acetate extract increased from 100 to 200 *µ*g/mL. The assay is based on the ability of the proteases to hydrolyse the protein in the agar. The protease activity may, however, be expressed in terms of proteolytic activity (*P*_*z*_) values as shown in Table 2. A strong proteolytic activity is observed for the positive control (*P*_*z*_=0.62) while moderate (*P*_*z*_=0.84) and weak (*P*_*z*_=0.94) proteolytic activities are observed in the presence of 100 *µ*g/mL and 200 *µ*g/mL, respectively, of the ethyl acetate extract. There was no growth, haloes, or proteolytic activity observed for the negative control.

## 4. Discussion

Different percentage yields of extracts were observed for total extractions and serial exhaustive extractions. These differences may be attributed to the different polarities of the solvents used [19] and the solubility of the phytochemicals in the respective solvents. Differences in the structure of phytochemicals determine their solubility in solvents of different polarities [20]. Higher percentage recovery of extractable compounds is observed mostly from polar solvents such as methanol and ethanol: water (50 : 50 v/v) for both total and serial exhaustive extractions. This is in contrast to nonpolar solvents and solvents of intermediate polarity such as hexane, DCM, acetone, and ethyl acetate. The higher yields obtained from polar solvents may be due to higher solubility of extractable bioactive components such as carbohydrates and protein [20]. Therefore, these observations may suggest that the *O. reticulata* root barks are rich in polar phytochemical compounds.

All the extracts demonstrated anti-*S. aureus* activity. The difference in antimicrobial activities observed from the extracts is likely due to the differences in the concentrations of antimicrobial compounds within the extracts. The higher the concentration of the antimicrobial compounds in the extracts, the greater the antimicrobial activity of the extract. The most potent extract was the ethyl acetate extract with an MIC of 100 *µ*g/mL. This means that, at a concentration of 100 *µ*g/ml of the ethyl acetate extract, there was complete inhibition of the growth of *S. aureus*. This observation suggests that the ethyl acetate extract had a higher concentration of antimicrobial compounds as compared to the other extracts. Ethyl acetate is a solvent of intermediate polarity and is capable of extracting slightly polar and nonpolar compounds. Phytochemical analysis of *O. reticulata* has revealed that the plant contains compounds such as essential oils, anacardic acids, ginkgolic acids, tannins, and flavonoids [21]. These compounds were shown to be extractable using ethyl acetate as an extraction solvent and have antimicrobial properties [21]. Therefore, these compounds may also be present in our sample and were responsible for the antibacterial activity that was observed.

Essential oils consist of terpenes, terpenoids, and aromatic compounds [22]. Some parts of these compounds are hydrophobic and some may be hydrophilic depending on their structure. The essential oils found in *O. reticulata* include tirucallane triterpenes and *a*-elemolic acid esters [21]. Essential oils have been shown to have an inhibitory activity against Gram-positive cocci such as MRSA. The peptidoglycan layer of Gram-positive bacteria allows hydrophobic substances such as essential oils to pass and reach the internal environment [23]. The mechanism of action of essential oils against Gram-positive bacteria is suggested to be through disruption of the cell wall and cytoplasmic membranes, cytoplasm coagulation, and alteration of the permeability and functions of the lipid bilayer [24].

Anacardic acids inhibit the growth of *S. aureus* through several mechanisms. Firstly, anacardic acids disrupt the membranes of *S. aureus* by acting as surfactants [25]. Secondly, amphipathic anacardic acids are capable of inhibiting bacterial respiration. This is through interaction with the electron transport chain system and ATPases. Thirdly, anacardic acids have been reported to have a chelating effect on metal ions such as Fe^2+^ and Cu^2+^ [26]. This reduces the availability of the ions to the bacteria. Fourthly, anacardic acids have an inhibitory activity towards *ß*-lactamase which is responsible for *ß*-lactam antibiotic resistance [27]. Lastly, anacardic acids have been reported to inhibit the synthesis of lipids in bacterial cells. The mechanism is through inhibition of glycerol-3-phosphate dehydrogenase enzyme [28]. Ginkgolic acids also affect the growth of *S. aureus*. Studies on the antibacterial mechanisms of ginkgolic acids revealed that they could affect the activity of numerous enzymes responsible for the survival of the bacteria such as protein phosphatases, lipoxygenases, and histone acetyltransferases [29, 30]. Furthermore, in another study, it was revealed that ginkgolic acids could inhibit DNA replication, RNA transcription, and protein synthesis in vivo in gram-positive bacteria [31]. Tannins have been reported to be bacteriostatic or bactericidal against *S. aureus*. The astringent properties of tannins may induce complexation with enzymes or substrates [32]. Many bacterial enzymes are inhibited when mixed with tannins. Furthermore, tannins affect bacterial cell membranes. Flavonoids have also been reported to possess interesting antibacterial mechanisms against *S. aureus.* These may also account for the effect of the ethyl acetate extract on the growth of *S. aureus* observed. Flavonoids affect the growth of *S. aureus* by inhibiting DNA, RNA, and protein synthesis. The proposed mechanism of this action was through the intercalation of flavonoids with nucleic acids [33]. The inhibition of *S. aureus* growth by flavonoids is through membrane disruption as they have been reported to cause potassium leakage in *S. aureus* cells thereby reducing their viability [34]. Flavonoids also disrupt *S. aureus* cell membranes through the disordering of membrane lipids and through altering the fluidity in hydrophilic and hydrophobic regions of the cell membranes [35].

The ethyl acetate extract was used to screen for the production of extracellular proteases by *S. aureus*. The zones of clearance are directly proportional to the number of extracellular proteases produced by *S. aureus.* A larger zone of clearance observed for the positive control is because there was no extract supplemented into the media. Therefore, there was no inhibition of the production of extracellular proteases by *S. aureus.* The zone of clearance significantly decreased in the presence of the ethyl acetate extract. The sizes of the zones of clearance are directly proportional to the concentration of the ethyl acetate extract. The zone of clearance observed at 100 *µ*g/mL of the ethyl acetate extract is larger in size compared to that at 200 *µ*g/mL of the ethyl acetate extract. This indicates the effectiveness of the ethyl acetate extract in inhibiting the production of extracellular proteases by *S. aureus* as its concentration increases.

The effectiveness of the ethyl acetate extract to inhibit the production of extracellular proteases can also be explained in terms of proteolytic activity values. This parameter is used to quantify the extent to which the extracellular proteases produced hydrolyse the protein within the agar media. The greater the *P*_*z*_ value, the weaker the proteolytic activity of the extracellular proteases. The proteolytic activity gets weaker as the concentration of the ethyl acetate extract increases from 0 *µ*g/mL (positive control) to 200 *µ*g/mL. This shows that, at higher concentrations of the ethyl acetate extract, the amount of extracellular proteases produced by *S. aureus* is low. Fewer proteases are present to hydrolyse the protein in the agar media. As a result, this demonstrates the effectiveness of the ethyl acetate extract in inhibiting the production of extracellular proteases.

The BCG-skim milk agar plates allow for the qualitative determination of protease activity. The BCG reagent binds to the unhydrolysed protein in the skim milk agar plates. The reagent is pH-dependent and shows a greenish-blue colour in the presence of unhydrolysed protein [18]. In the presence of extracellular proteases from *S. aureus*, the protein in the media is hydrolysed to amino acids. This lowers the pH and liberates the BCG reagent resulting in a distinct clear zone that is observed.

Inhibition of the production of *S. aureus* extracellular proteases by the ethyl acetate extract from *O. reticulata* root barks can be attributed to several mechanisms. *O. reticulata* has been reported to contain tirucallane triterpenes [21]. These may also be present in the ethyl acetate extract. Triterpenes and triterpenoids have been associated with the downregulation of the production of secreted proteins [36]. While the exact mechanism of action of this phenomenon is still unknown, several suggestions have been put forward. For example, it is known that triterpenes have the potential to inhibit protease dimerization [37]. This is due to the molecular size of triterpenes which allow them to fit into the hydrophobic interface of the relaxed protease monomer. In the process, the production of functional proteases may be inhibited. Furthermore, hydrogen bonding is possible between the proteases and the hydroxyl/carboxyl groups in the triterpenes scaffold [38].

The production of bacterial virulence factors is regulated by the quorum sensing system. Quorum sensing (QS) refers to the way microorganisms regulate their behaviour through sending and receiving chemical signals (autoinducers). *S. aureus* uses QS to regulate the production of virulence factors including the extracellular proteases. Some phytochemicals have been shown to have an inhibitory effect on the QS system of *S. aureus.* Inhibitors that target virulence factor production by the QS system affect the transcription and translation of the virulence factors [39]. Therefore, this suggests that the ethyl acetate extract from *O. reticulata* root barks may contain phytochemicals that inhibit or quench the QS system, thereby, preventing extracellular protease production.

## 5. Conclusion

The root bark extracts from *O. reticulata* inhibited the growth of *S. aureus*. The most potent inhibitor of the growth of *S. aureus* was the ethyl acetate extract and it was found to be bacteriostatic. The ethyl acetate extract inhibited extracellular protease production by *S. aureus.* Root bark extracts from *O. reticulata*, therefore, contain promising phytochemical compounds that may be able to combat infections caused by *S. aureus*. This may minimize the severity of *S. aureus* infections and the development of antibiotic-resistant strains of *S. aureus*.

## Figures and Tables

**Table 1:** Percentage yields and inhibitions of *O. reticulata* root bark extracts.

Extraction solvent	Colour and consistency after drying	Weight of extract (g)	Percentage of yield	Percentage inhibition
Ethanol : water	Dark brown, solid	2.906	15	32
DCM : methanol	Dark brown, solid	1.336	7	29
Hexane	Pale yellow, viscous	1.908	10	62
DCM	Brown, solid	0.861	5	93
Acetone	Brown, solid	1.250	7	54
Ethyl acetate	Dark brown, viscous	0.390	3	99
Methanol	Dark brown, solid	1.502	10	67
Ethanol	Brown, solid	0.905	7	50
Water	Brown, solid	0.195	2	56

**Table 2:** The proteolytic activities of the extracellular proteases produced from *S. aureus* on BCG-skim milk agar plates: A, B, C, and D.

Plate	Colony diameter (mm)	Colony diameter + halo (mm)	*P*_*z*_ value	Reference *P*_*z*_ value	Proteolytic activity
A	10	16	0.62 < 0.69	Very strong	
B	11	13	0.84	0.80–0.89	Mild
C	8	8.5	0.94	0.90–0.99	Weak
D	—	—	—	—	—

*P*_*z*_ value is the ratio of the diameter of the colony to the total diameter of the colony plus the zone of hydrolysis.

## Data Availability

The datasets used and/or analyzed during the current study are available from the corresponding author on reasonable request.

## Abbreviations

DCM: Dichloromethane
MIC: Minimum inhibitory concentration
MBC: Minimum bactericidal concentration
MTT: 3-(4, 5-Dimethylthiazol-2-yl)-2, 5-diphenyltetrazolium bromide
DMSO: Dimethyl sulfoxide
NCTC: National Collection of Type Cultures
MRSA: Methicillin-resistant *Staphylococcus aureus*
MSSA: Methicillin-susceptible *Staphylococcus aureus*
BCG: Bromocresol green
OD: Optical density
QS: Quorum sensing
CCU: Critical care unit.
